# Supplementary material for: Comparing copy-number profiles under multi-copy amplifications and deletions
Source: BMC Genomics. 2020 Apr 16;21(Suppl 2):198. doi: 10.1186/s12864-020-6611-3 (PMC7160889; doi:10.1186/s12864-020-6611-3)
Supplement: Supplementary file 1 — Additional file 1 Supplementary file S33-S1.pdf contains all the missing proofs. [file 12864_2020_6611_MOESM1_ESM.pdf]

Comparing copy-number profiles under multi-copy amplifications and deletions Supplementary Material I Additional proofs

Lemma 1. Let $\boldsymbol{u}, \boldsymbol{v}$ be two CNPs with no null positions. If $\boldsymbol{u} - \boldsymbol{v}$ contains a staircase $[a, b]$ of length k, then $d_f(\boldsymbol{u}, \boldsymbol{v}) \geq k$ for any unit-cost function f.

Proof of Lemma 1. We use induction on the length k of the staircase. When $k = 1$, it is obvious that $d_f(\boldsymbol{u}, \boldsymbol{v}) \geq 1$ as we need to apply at least one event on $\boldsymbol{u}$. Now assume the lemma is true for values less than k, and that for two given vectors $\boldsymbol{u}^*, \boldsymbol{v}^*$ such that $\boldsymbol{u}^* - \boldsymbol{v}^*$ contains a staircase of length $k' < k$, $d_f(\boldsymbol{u}^*, \boldsymbol{v}^*) \geq k'$. Suppose that two given CNPs $\tilde{\boldsymbol{u}}$ and $\tilde{\boldsymbol{v}}$ contain a staircase of length k in interval $[a, a + k - 1]$ in their difference vector. Let $\boldsymbol{u} = (\tilde{u}_a, \dots, \tilde{u}_{a+k-1})$ and $\boldsymbol{v} = (\tilde{v}_a, \dots, \tilde{v}_{a+k-1})$. By Proposition 1, $d_f(\tilde{\boldsymbol{u}}, \tilde{\boldsymbol{v}}) \geq d_f(\boldsymbol{u}, \boldsymbol{v})$ since we have only removed some positions. Moreover, $\boldsymbol{u} - \boldsymbol{v}$ consists of a staircase in interval $[1, k]$. Let $E = (e_1, \dots, e_l)$ be a sequence of length $l := d_f(\boldsymbol{u}, \boldsymbol{v})$ satisfying $\boldsymbol{u}\langle E \rangle = \boldsymbol{v}$ (note that $l = d_f(\boldsymbol{u}, \boldsymbol{v})$ because f is unit-cost). If we show that $d(f, \mathbf{u})\boldsymbol{v} = l \geq k$, then we are done. Let us assume, for the sake of contradiction, that $l < k$. Under this assumption and the inductive hypothesis, we show two properties on E.

Property 1: no amplification of E affects position k, the last position of $\boldsymbol{u}$. Assume otherwise, and suppose that some amplification event $\hat{e} \in E$ affects interval $[c, k]$ for some $c \in [k]$. By Proposition 2, we may take an amp-first reordering of E and assume that $\hat{e} = e_1$ is the first event of E. Let $\hat{\boldsymbol{u}} := \boldsymbol{u}\langle \hat{e} \rangle$, and notice that $\hat{\boldsymbol{u}} - \boldsymbol{v}$ must contain a staircase of length $k-1$ in interval $[1, k-1]$. We may apply our inductive hypothesis and we reach a contradiction, since we get $k - 1 \leq d_f(\hat{\boldsymbol{u}}, \boldsymbol{v}) = d_f(\boldsymbol{u}, \boldsymbol{v}) - 1 \leq (k - 1) - 1$ (the latter by the assumption that $d_f(\boldsymbol{u}, \boldsymbol{v}) = l < k$).

Property 2: all events of E affect at least one position in $[1, k - 1]$. We use a similar idea. Assume that some event $\hat{e}$ of E does not affect any position in $[1, k - 1]$, i.e. it only affects position k and therefore we may write $\hat{e} = (k, k, b)$. By Property 1, $\hat{e}$ must be a deletion. Moreover, since no amplification ever affects position k, $\hat{\boldsymbol{u}} := \boldsymbol{u}\langle \hat{e} \rangle$ does not have 0 at position k, and we may further assume that $\hat{e}$ is the first event of E (since applying the other events will never

make position k drop below 0). In other words, $d_f(\boldsymbol{u}, \boldsymbol{v}) = d_f(\hat{\boldsymbol{u}}, \boldsymbol{v}) + 1$. But then $\hat{\boldsymbol{u}}$ has a staircase in interval $[1, k-1]$ and by the same arguments as above, $k - 1 \leq d_f(\hat{\boldsymbol{u}}, \boldsymbol{v}) = d_f(\boldsymbol{u}, \boldsymbol{v}) - 1 \leq (k-1) - 1$, again a contradiction.

So far, we know that only deletions affect position k (Property 1), and all these deletions also affect position $k-1$ (Property 2). Because $u_{k-1} - v_{k-1} < u_k - v_k$ and $v_{k-1} > 0$, this implies that some amplification event $\hat{e}$ must affect position $k-1$ (otherwise, applying only the deletion events affecting position k on position $k-1$ would make position $k-1$ drop below v_{k-1}). Let us assume, again using Proposition 2, that $\hat{e}$ is the first event of E, i.e. $e_1 = \hat{e}$. We use the same trick for a third time. That is, let $\hat{\boldsymbol{u}} := \boldsymbol{u}\langle \hat{e} \rangle$ and notice that $\hat{\boldsymbol{u}}$ has a staircase in interval $[1, k-1]$. Once again we obtain $k - 1 \leq d_f(\hat{\boldsymbol{u}}, \boldsymbol{v}) = d_f(\boldsymbol{u}, \boldsymbol{v}) - 1 \leq (k-1) - 1$. This contradiction forces us to conclude that $l < k$ is false, which proves the lemma. □

Lemma 2. Let $\boldsymbol{u}$ and $\boldsymbol{v}$ be two CNPs with no null positions and let f be any unit-cost function. If $\boldsymbol{u} - \boldsymbol{v}$ contains a staircase in interval $[1, k]$ and $d_f(\boldsymbol{u}, \boldsymbol{v}) = k$, then there exists a smooth sequence transforming $\boldsymbol{u}$ into $\boldsymbol{v}$.

Proof of Lemma 2. We prove the lemma by induction over k. As a base case, the statement is easy to see when $k = 1$ since a single step can only removed by a deletion, which is smooth. So assume $k > 1$ and that for any $\boldsymbol{u}', \boldsymbol{v}'$ such that $d_f(\boldsymbol{u}', \boldsymbol{v}') = k - 1$ and such that $\boldsymbol{u}' - \boldsymbol{v}'$ have a staircase of length $k-1$ in $[1, k-1]$, there is an optimal smooth sequence transforming $\boldsymbol{u}'$ into $\boldsymbol{v}'$.

Let E be any sequence of k events such that $\boldsymbol{u}\langle E \rangle = \boldsymbol{v}$. If E is smooth, then we are done so assume otherwise. The proof is divided in two parts. Assuming the inductive hypothesis, we first show that there is an optimal sequence $\hat{E}$ containing only deletions such that $\boldsymbol{u}\langle \hat{E} \rangle = \boldsymbol{v}$. These deletions are not necessarily smooth. We complete the induction in a second step, where we convert this deletion sequence into a smooth one. For the remainder of the proof, we will denote $\boldsymbol{w} := \boldsymbol{u} - \boldsymbol{v}$.

Part 1: proof that $\boldsymbol{u}$ can be transformed into $\boldsymbol{v}$ using only deletions. Assume that $E = (e_1, \ldots, e_k)$ contains some amplification, otherwise we are done proving our first step. We first claim that only deletions affect positions k to n, inclusively. To see this, assume on the contrary that $e_i = (a, b, \delta)$ is an amplification where $b \geq k$. By Proposition 2, we may assume that $e_i = e_1$. But $\boldsymbol{u}\langle e_1 \rangle$ still has a staircase in interval $[1, k]$, and by Lemma 1, $d_f(\boldsymbol{u}, \boldsymbol{v}) \geq k$. This is a contradiction since e_1 should reduce the distance to from $\boldsymbol{u}$ to $\boldsymbol{v}$. Hence our claim holds.

We now claim that, on the other hand, some amplification in E affects position $k-1$. This is clearly true if every deletion affecting position k also affects position $k-1$. Indeed, we have $w_{k-1} < w_k$ and without an amplification on $k-1$ it would be impossible that position $k-1$ becomes equal to $v_{k-1} > 0$. Thus if we suppose that no amplification affects position $k-1$, there must be some

deletion $e_i = (k, h, d)$ that affects position k but not $k-1$, where here $h \geq k$. Let $\boldsymbol{u}' := \boldsymbol{u}\langle e_i \rangle$. Since no amplification affects any position in $[k, h]$, $\boldsymbol{u}'$ has no position with value 0. Furthermore, $\boldsymbol{u}' - \boldsymbol{v}$ contains a staircase of length $k-1$ at $[1, k-1]$ and it is clear that $d_f(\boldsymbol{u}', \boldsymbol{v}) = k-1$. By induction, there is a (smooth) deletion sequence E' such that $\boldsymbol{u}'\langle E' \rangle = \boldsymbol{v}$. In that case, the sequence formed by e_i followed by E' transforms $\boldsymbol{u}$ into $\boldsymbol{v}$ and has only deletions, which is what we want. Thus we may assume that our claim saying that some amplificatio affects $k-1$ holds.

Moving on, let $e_i = (a, k-1, \delta)$ be an amplification in E that affects position $k-1$ (but not k). Our previous claims show that e_i exists. By Proposition 2, we may assume that $e_1 = e_i$. Let $\boldsymbol{u}' := \boldsymbol{u}\langle e_1 \rangle$ and $\boldsymbol{w}' := \boldsymbol{u}' - \boldsymbol{v}$. Then $\boldsymbol{w}'$ has a staircase of length $k-1$ in interval $[1, k-1]$ and $d_f(\boldsymbol{u}', \boldsymbol{v}) = k-1$. Moreover, the differences in value between the steps have not changed, except at position a. Formally, for each $i \in [k-1] \setminus \{a\}$, $w'_i - w'_{i-1} = w_i - w_{i-1}$ and $w'_a - w'_{a-1} = w_a - w_{a-1} + \delta$.

By induction, $\boldsymbol{u}'\langle E' \rangle = \boldsymbol{v}$ for some smooth deletion sequence $E' = (e'_1, \ldots, e'_{k-1})$. Here for each $i \in [k-1]$, $e'_i = (i, b_i, w'_{i-1} - w'_i)$ for some $b_i \geq k-1$. Let $(i_1, b_{i_1}, d_{i_1}), \ldots, (i_l, b_{i_l}, d_{i_l})$ be the deletion events of E' that affect position k, $i_1 < i_2 < \ldots < i_l$. We distinguish two cases.

Case 1: $a \notin \{i_1, \ldots, i_l\}$. Then the event $(a, b_a, w'_{a-1} - w'_a)$ of E' does not affect position k, meaning that $b_a = k-1$ (by smoothness). Consider the sequence E'' obtained from E' by replacing the event $(a, k-1, w'_{a-1} - w'_a)$ by the event $(a, k-1, w_{a-1} - w_a)$. Since $\boldsymbol{u}'\langle E' \rangle - \boldsymbol{v}$ has a 0 everywhere and $w'_a - w'_{a-1} = w_a - w_{a-1} + \delta$, it follows that $\boldsymbol{u}'\langle E'' \rangle - \boldsymbol{v}$ has value 0 everywhere, except at positions from a to $k-1$ where it has value δ. But then, the only difference between $\boldsymbol{u}$ and $\boldsymbol{u}'$ is that positions a to $k-1$ are increased by δ. Thus $\boldsymbol{u}\langle E'' \rangle - \boldsymbol{v}$ has a value of 0 everywhere (and $\boldsymbol{u}$ never drops below 0, due to the smoothness of E'). This means that $\boldsymbol{u}\langle E'' \rangle = \boldsymbol{v}$, which is a contradiction since E'' has $k-1$ events.

Case 2: $a = i_h$ for some $h \in [l]$. Then the deletion of E' starting at a is $(a, b_a, -(w'_a - w'_{a-1})) = (a, b_a, w_{a-1} - w_a - \delta)$ and affects position k, i.e. $b_a \geq k$. Consider the sequence E'' obtained from E' by replacing the event $(a, b_a, w_{a-1} - w_a - \delta)$ by $(a, b_a, w_{a-1} - w_a)$. Then $\boldsymbol{u}'\langle E'' \rangle - \boldsymbol{v}$ has a 0 everywhere, except at positions from a to b_a where it has value δ. Also, $\boldsymbol{u}\langle E'' \rangle - \boldsymbol{v}$ has a 0 everywhere, except at positions from k to b_a where it has value δ. We can apply the deletion $(k, b_a, -\delta)$ to $\boldsymbol{u}\langle E'' \rangle$ to obtain $\boldsymbol{v}$. Since E'' has $k-1$ events, this yields a sequence of k deletions transforming $\boldsymbol{u}$ into $\boldsymbol{v}$.

This concludes the first part. That is, we have shown that if our inductive hypothesis holds, then some deletion sequence of length k transforms $\boldsymbol{u}$ into $\boldsymbol{v}$.

Part 2: construction of a smooth sequence. Now let $\hat{E} = (\hat{e}_1, \ldots, \hat{e}_k)$ be a sequence of k deletions transforming $\boldsymbol{u}$ into $\boldsymbol{v}$, which exists by Part 1. Let $(1, b, \delta)$ be any deletion affecting position 1. Since $\hat{E}$ contains only deletions,

it is safe to assume that $\hat{e}_1 = (1, b, \delta)$. Let $\boldsymbol{u}' := \boldsymbol{u}\langle \hat{e}_1 \rangle$ and $\boldsymbol{w}' := \boldsymbol{u}' - \boldsymbol{v}$. If $-\delta < w_1$, then $\boldsymbol{w}'$ contains a staircase of length k and we reach a contradiction since this implies $d_f(\boldsymbol{u}', \boldsymbol{v}) \geq k$. If $-\delta > w_1$, then $w'_1 < 0$ and position 1 can never have the same value as v_1 since $\hat{E}$ has only deletions. We deduce that $-\delta = w_1$.

It follows that $\boldsymbol{u}'$ has a staircase of length $k-1$ in positions $[2, k]$. No event of $\hat{E}$ can affect position 1 after e_1, so we can ignore this position in $\boldsymbol{u}'$ and $\boldsymbol{w}'$. That is, suppose we remove position 1 from $\boldsymbol{u}'$ and $\boldsymbol{v}$, yielding two vectors $\boldsymbol{u}''$ and $\boldsymbol{v}'$ of length $n-1$. Let $\boldsymbol{w}'' := \boldsymbol{u}'' - \boldsymbol{v}'$. Then $\boldsymbol{w}''$ has a staircase of length $k-1$ in interval $[1, k-1]$. This allows us to use induction, so that there is a smooth sequence $\hat{E}''$ of length $k-1$ transforming $\boldsymbol{u}''$ into $\boldsymbol{v}'$. This easily translates into a sequence $\hat{E}'$ transforming $\boldsymbol{u}'$ into $\boldsymbol{v}$: we just "shift" every event to the right to account for position 1 in $\hat{E}'$. To be specific, we replace any event (s, t, ϵ) from $\hat{E}''$ by the event $(s+1, t+1, \epsilon)$ in $\hat{E}'$. Since $\hat{E}''$ is smooth, then we can write $\hat{E}' = ((2, b_2, \epsilon_2), \ldots, (k, b_k, \epsilon_k))$ where, for each $i \in \{2, \ldots, k\}$, $b_i \geq k$ and $d_i = w'_i - w'_{i-1}$.

We have not shown smoothness yet, because $\hat{e}_1$ might not affect the whole $[1, k]$ interval as we wish. If indeed $\hat{e}_1$ affects position k, i.e. if $b \geq k$, then it is easy to see that applying $\hat{e}_1$ followed by $\hat{E}'$ is a smooth sequence transforming $\boldsymbol{u}$ into $\boldsymbol{v}$. Thus we may assume that $b < k$. Observe that $w'_i - w'_{i-1} = w_i - w_{i-1}$ for all $i \in \{2, \ldots, k\} \setminus \{b+1\}$, because $w'_{b+1} - w'_b = w_{b+1} - w_b + w_1$ (recall that $-\delta = w_1$). Let $(b+1, b', w_b - w_{b+1} - w_1)$ be the deletion of $\hat{E}'$ that starts at position b, where $b' \geq k$ by smoothness. Suppose that we replace it with the deletion $(b+1, b', w_b - w_{b+1})$ in $\hat{E}'$, yielding an alternate sequence $\tilde{E}$. Then $\boldsymbol{u}'\langle \tilde{E} \rangle - \boldsymbol{v}$ has a 0 everywhere, except at positions $b+1$ to b' where it has value w_1. This means that if in $\hat{E}$, we replace $\hat{e}_1$ by $\tilde{e} = (1, b', -w_1)$ and follow it by $\tilde{E}$, we obtain a sequence transforming $\boldsymbol{u}$ into $\boldsymbol{v}$. Now, let $\tilde{\boldsymbol{u}} := \boldsymbol{u}\langle \tilde{e} \rangle$. If we remove position 1 from $\tilde{\boldsymbol{u}}$ (recalling that $\tilde{u}_1 = v_1$) and from $\boldsymbol{v}$, we obtain a CNP with a staircase at $[1, k-1]$. Applying induction, we get a smooth sequence $\tilde{E}''$ which we can modify into $\tilde{E}'$ to make it applicable to $\boldsymbol{u}$ (just as we did from $\hat{E}''$ to $\hat{E}'$). It is then straightforward to see that $\tilde{e_1}$ followed by $\tilde{E}'$ is a smooth deletion sequence turning $\boldsymbol{u}$ into $\boldsymbol{v}$. □

Theorem 1. The CNP-transformation problem is strongly NP-hard for any deletion-permissive unit-cost function, even if the CNPs have no null positions.

Proof of Theorem 1. From a 3-partition instance $S = \{s_1, \ldots, s_n\}$, construct $\boldsymbol{u}$ and $\boldsymbol{v}$ as follows. First define $K := 100n$ and, for all $i \in [n]$, put $p_i := \sum_{j=1}^{i} s_j$, the idea being that p_i and p_{i-1} differ by an amount of s_i. Then put $\boldsymbol{v}$ as a vector containing only 1s. For $\boldsymbol{u}$, construct it by adding one position at a time from left to right: first insert the values $i + 1 + Kp_i$ for $i = 1..n$, and then the values $i(Kt+3)+1$ for $i = m..1$. That is, let

$$\boldsymbol{v} = (1, 1, \ldots, 1)$$
$$\boldsymbol{u} = (2 + Kp_1, 3 + Kp_2, \ldots, n + 1 + Kp_n, m(Kt+3)+1, \ldots, (Kt+3)+1)$$

This can be done in polynomial time in n (in particular, each p_i is polynomial). Observe that we have

$$\boldsymbol{w} = (1 + Kp_1, \ldots, n + Kp_n, m(Kt + 3), \ldots, Kt + 3)$$

In particular, $\boldsymbol{w}$ has a staircase in interval $[1, n]$, followed by a decreasing staircase in interval $[n + 1, n + m]$. By Lemma 1, we know that $d_f(\boldsymbol{u}, \boldsymbol{v}) \geq n$. We will show that S is a YES-instance to 3-partition if and only if $d_f(\boldsymbol{u}, \boldsymbol{v}) = n$.

($\Rightarrow$): Suppose that there exists m triplets $S_1, \ldots, S_m$ such that $\sum_{s' \in S_i} s' = t$ for all $i \in [m]$. We may assume that each $s_i \in S$ is distinguishable, so that for each s_i there is a unique k such that $s_i \in S_k$. We construct a sequence $E = (e_1, \ldots, e_n)$ of n deletions such that $\boldsymbol{u}\langle E \rangle = \boldsymbol{v}$. For each $i \in [n]$, put $e_i = (i, n + k, w_{i-1} - w_i)$, where k if the unique integer such that $s_i \in S_k$. Note that the e_i events are allowed because f is deletion-permissive (this is actually the only place where we need this assumption). One can check that E is a smooth deletion sequence and it is clear that positions 1 to n become equal to 1 after applying E on $\boldsymbol{u}$. Now consider the events that end at position $n + k$, $k \in [m]$. For each $s_i \in S_k$, there is such an event that decreases all the positions $n + 1$ to $n + k$ by $w_i - w_{i-1} = Ks_i + 1$. We get $\sum_{s_i \in S_k}(Ks_i + 1) = Kt + 3$. Since this is true for every position from $n + 1$ to $n + m$, the total decrease for a position $k \in [m]$ will be $\sum_{j=k}^{m} Kt + 3 = (m + 1 - k)Kt + 3$, which is exactly w_{n+k}. Hence $\boldsymbol{u}\langle E \rangle = \boldsymbol{v}$.

($\Leftarrow$): Assume that $d_f(\boldsymbol{u}, \boldsymbol{v}) = n$. Let $E = (e_1, \ldots, e_n)$ be an optimal sequence of events transforming $\boldsymbol{u}$ into $\boldsymbol{v}$. By Lemma 2, we may assume that E is smooth. Thus each e_i is a deletion of the form $(i, b_i, w_{i-1} - w_i) = (i, b_i, -(Ks_i + 1))$, where $b_i \in [n, n + m]$. Let us define $S_k := \{s_i : b_i = n + k\}$. We claim that $\sum_{s_i \in S_k}(Ks_i + 1) = Kt + 3$. For $k = m$, this must be true since $w_{n+m} = Kt + 3$. For $k < m$, we have the difference $w_{n+k} - w_{n+k+1} = Kt+3$. This means that the deletions that affect position $n+k$ but not $n+k+1$ (i.e. those with $b_i = n+k$) must incur a total decrease of exactly $Kt + 3$, as claimed. We now argue that $|S_k| = 3$ for each $k \in [m]$. Notice that $\sum_{s_i \in S_k}(Ks_i + 1) = K \sum_{s_i \in S_k} s_i + |S_k| = Kt + 3$. If $\sum_{s_i \in S_k} s_i = t$, then $|S_k| = 3$. Otherwise, by isolating the $|S_k|$ term above, it is not hard to deduce that $|S_k| \geq K$. However, this is impossible since $|S_k| \leq n$ but $K > n$. We have therefore shown that $|S_k| = 3$, which in turn implies that $\sum_{s_i \in S_k} s_i = t$.Therefore S is a YES instance. □

Lemma 3. Let $\boldsymbol{u}, \boldsymbol{v}$ be two distinct CNPs with no null positions, and let $\boldsymbol{w} := \boldsymbol{u} - \boldsymbol{v}$. Then for any unit-cost function f, $d_f(\boldsymbol{u}, \boldsymbol{v}) \geq \lceil (|F_{\boldsymbol{w}}| - 1)/2 \rceil$.

Proof of Lemma 3. We prove the Lemma by induction on $d_f(\boldsymbol{u}, \boldsymbol{v})$. As a base case, when $d_f(\boldsymbol{u}, \boldsymbol{v}) = 1$, then $F_{\boldsymbol{w}}$ has 3 flat intervals: the extreme ones and the flat interval that gets affected in the single event transforming $\boldsymbol{u}$ into $\boldsymbol{v}$ (recall that we have artificial positions $w_0 = 0$ and $w_{n+1} = 0$, which guarantee that there are always two extreme intervals plus another one somewhere in $[i1, n]$). The statement is clearly true in this case, as $\lceil |F_{\boldsymbol{w}}| - 1)/2 \rceil = 1$.

Now assume that the Lemma holds for any pair of CNPs $\boldsymbol{u}', \boldsymbol{v}'$ satisfying $d_f(\boldsymbol{u}', \boldsymbol{v}') < d_f(\boldsymbol{u}, \boldsymbol{v})$. Let $E = (e_1, \ldots, e_k)$ be an optimal sequence of events

such that $\boldsymbol{u}\langle E\rangle = \boldsymbol{v}$. Let $\hat{\boldsymbol{u}} := \boldsymbol{u}\langle e_1\rangle$ and $\hat{\boldsymbol{w}} := \hat{\boldsymbol{u}} - \boldsymbol{v}$. Let $e_1 = (c, d, x)$, where x could be negative in case of a deletion. Let $F'_{\boldsymbol{w}} = \{[a, b] \in F_{\boldsymbol{w}} : [a, b] \cap [c, d] \neq \emptyset\}$ be the affected flat intervals. Assume that $F'_{\boldsymbol{w}}$ has $l \geq 0$ intervals, say $F'_{\boldsymbol{w}} = \{[a_1, b_1], \ldots, [a_l, b_l]\}$, and that they are ordered so that $b_i + 1 = a_{i+1}$ for each $i \in [l-1]$.

First consider $[a_i, b_i]$ with $2 \leq i \leq l-1$. Note that $[a_i, b_i]$ cannot be an extreme flat interval in $\boldsymbol{w}$. We claim that $[a_i, b_i]$ must still be a non-extreme flat interval in $\hat{\boldsymbol{u}}$. To see this, observe that $\hat{\boldsymbol{w}}_{a_i-1} = \boldsymbol{w}_{a_i-1} + x$ and $\hat{\boldsymbol{w}}_{a_i} = \boldsymbol{w}_{a_i} + x$. Since $\boldsymbol{w}_{a_i-1} \neq \boldsymbol{w}_{a_i}$ by maximality, we have $\hat{\boldsymbol{w}}_{a_i-1} \neq \hat{\boldsymbol{w}}_{a_i}$. By a similar argument, $\hat{\boldsymbol{w}}_{b_i+1} \neq \hat{\boldsymbol{w}}_{b_i}$. And because all values in $[a_i, b_i]$ have changed by the same amount x, $[a_i, b_i]$ is a (maximal) flat interval (note that we need the assumption of no null positions to argue that all positions change by the same amount). Moreover, $[a_i, b_i]$ cannot be extreme. If instead $[a_i, b_i]$ was in the extreme interval containing w_0, then we would have $\hat{\boldsymbol{w}}_h = 0$ for all $0 \leq h \leq b_i$. In particular, this would imply $\hat{\boldsymbol{w}}_{a_i-1} = \hat{\boldsymbol{w}}_{a_i}$, contrary to what we just argued. The same occurs if we assume that $[a_i, b_i]$ is part of the extreme interval containing w_{n+1}.

Now consider any flat interval $[a, b] \in F_{\boldsymbol{w}} \setminus F'_{\boldsymbol{w}}$. It is easy to see that $[a, b]$ is still a flat interval in $\hat{\boldsymbol{w}}$, unless perhaps if $b + 1 = a_1$ or $a - 1 = b_l$. In these cases, it is possible that $\hat{\boldsymbol{w}}_b = \hat{\boldsymbol{w}}_{a_1}$ and/or $\hat{\boldsymbol{w}}_a = \hat{\boldsymbol{w}}_{b_l}$. These have the effect of "merging" two flat intervals, effectively eliminating $[a_1, b_1]$ and/or $[a_l, b_l]$ (note that the argument also holds when $[a_1, b_1]$ or $[a_l, b_l]$ become part of an extreme interval). Since every flat interval except these two stays in $\hat{\boldsymbol{w}}$, it follows that $|F_{\hat{\boldsymbol{w}}}| \geq |F_{\boldsymbol{w}}| - 2$. Then using induction,

$$d_f(\boldsymbol{u}, \boldsymbol{v}) - 1 = d_f(\hat{\boldsymbol{u}}, \boldsymbol{v}) \geq \lceil (|F_{\boldsymbol{w}}| - 3)/2 \rceil = \lceil (|F_{\boldsymbol{w}}| - 1)/2 \rceil - 1$$

and it follows that $d_f(\boldsymbol{u}, \boldsymbol{v}) \geq \lceil (|F_{\boldsymbol{w}}| - 1)/2 \rceil$. □

Lemma 4. Suppose that $v_i = v_{i+1} = 0$ for some position i. Then removing position i or $i + 1$, whichever is smaller in $\boldsymbol{u}$, from $\boldsymbol{u}$ and $\boldsymbol{v}$ preserves the distance between $\boldsymbol{u}$ and $\boldsymbol{v}$. Formally, for any unit-cost function f, if $u_i \geq u_{i+1}$, then $d_f(\boldsymbol{u}, \boldsymbol{v}) = d_f(\boldsymbol{u}^{-\{i+1\}}, \boldsymbol{v}^{-\{i+1\}})$. Similarly if $u_{i+1} \geq u_i$, then $d_f(\boldsymbol{u}, \boldsymbol{v}) = d_f(\boldsymbol{u}^{-\{i\}}, \boldsymbol{v}^{-\{i\}})$.

Proof of Lemma 4. Assume that $u_i \geq u_{i+1}$ (the other case is identical). We know that $d_f(\boldsymbol{u}, \boldsymbol{v}) \geq d_f(\boldsymbol{u}^{-\{i+1\}}, \boldsymbol{v}^{-\{i+1\}})$, by Proposition 1. We consider the converse bound. Take any sequence $E = (e_1, \ldots, e_k)$ of events transforming $\boldsymbol{u}^{-\{i+1\}}$ into $\boldsymbol{v}^{-\{i+1\}}$. Modify E to transform $\boldsymbol{u}$ into $\boldsymbol{v}$ as follows: each event affects the same positions as before (including those that have shifted after reinserting $i+1$), but we ensure that every event affecting position i also affects position $i + 1$. To be formal, define $E' = (e'_1, \ldots, e'_k)$ as follows. If e_i increases interval $[a, b]$ by δ (which is possibly negative), then make e'_i increase interval $[a', b']$ by δ, where

$$a' = \begin{cases} a & \text{if } a \leq i \\ a+1 & \text{if } a > i \end{cases} \qquad b' = \begin{cases} b & \text{if } b < i \\ b+1 & \text{if } b \geq i \end{cases}$$

Aside from the new position i in $\boldsymbol{u}$ and $\boldsymbol{v}$, every position reaches the same value as before. Also because $u_i \geq u_{i+1}$, position $i+1$ reaches 0 after applying E' on $\boldsymbol{u}$. □

Lemma 5. Suppose $v_i = 0$ for some position i and that $w_{i-1} \geq w_i$ or $w_{i+1} \geq w_i$. Then $d_f(\boldsymbol{u}, \boldsymbol{v}) = d_f(\boldsymbol{u}^{-\{i\}}, \boldsymbol{v}^{-\{i\}})$ for any unit-cost function f.

Proof of Lemma 5. The proof is essentially the same as in Lemma 4. If, without loss of generality, $w_{i-1} \geq w_i$, we can take an event sequence from $\boldsymbol{u}^{-\{i\}}$ to $\boldsymbol{v}^{-\{i\}}$ and adapt it so that every event affecting position $i-1$ also affects position i. This guarantees that position i drops to 0. We omit the technical details. □

Finding good events in time $O(n \log n)$

We say that an event e is *good* if applying it on $\boldsymbol{u}$ reduces $|F_{\boldsymbol{w}}|$ by 2. Here we present the detailed version of our improved heuristic. The main algorithm that follows transforms $\boldsymbol{u}$ into $\boldsymbol{v}$ by making calls to the *findGoodEvent* subroutine, which is defined afterwards.

Data: vectors $\boldsymbol{u}, \boldsymbol{v}$
Result: Find a sequence that transforms $\boldsymbol{u}$ into $\boldsymbol{v}$
compute $\boldsymbol{w} := \boldsymbol{u} - \boldsymbol{v}$;
initialize empty sequence S;
for $u \neq v$ **do**
    if *findGoodEvent(**u**, **v**, **w**) returns* (i, j, x) **then**
        add (i, j, x) to S;
        for $k = i, ..., j$ **do**
            $u_k = \max u_k + x, 0$
    else
        find the first flat interval $[i, j]$ with $w_i \neq 0$;
        increase $u_i, \ldots u_j$ by $-w_i$;
        add $(i, j, -w_i)$ to S;
return S

Algorithm 1: Main algorithm

The algorithm *findGoodEvent* below can be implemented in time $O(n \log n)$. Our goal is to find a range of values $[i, j]$ that verifies $w_i - w_{i-1} = w_j - w_{j+1} := -\delta$. We further need that $\delta > 0$, or that $\delta < 0$ and $\forall k \in [i, j], u_k \geq -\delta :$ we can then apply the event (i, j, δ). To achieve this, the idea is simply to scan $\boldsymbol{w}$ from left to right. Each time we detect a change of $w_k - w_{k+1}$, we check if we encountered the same amount of change before at some position k' (this is $-\delta$ in the algorithm). If so, we can return the k, k' pair since it can be part of a good event. Otherwise, we map $\delta = w_{k+1} - w_k$ to position $k+1$ to store the fact that $k+1$ is the latest position that could be matched with a change of δ. The last line of the for loop ensures that if we match two positions $k' < k$, all positions in-between are sufficiently high to allow a deletion of amount δ.

Data: vectors $\boldsymbol{u}, \boldsymbol{v}, \boldsymbol{w}$
Result: Find an event that reduces $|F_{\boldsymbol{w}}|$ by 2
initialization of an empty dictionary R;
for $k = 1, ..., n-2$ **do**
  $\delta := w_{k+1} - w_k$;
  if $\delta == 0$ **then** continue ;
  if $-\delta \in R$ **then**
    return $(R[-\delta], k, \delta)$;
  else
    Set $R[\delta] = k + 1$;
    delete all the key/value pairs (x, y) in R with $u_k \leq x$;
return *no possible event*

Algorithm 2: findGoodEvent

We argue two components: that *findGoodEvent* does find a good event, if there is one, and that it can be implemented to take time $O(n \log n)$.

Proof that Algorithm *findGoodEvent* returns an event (i, j, δ) that reduces $|F_w|$ by 2 when it exists. Consider an output (i, j, δ). Due to the construction, we had $-\delta \in R$, which can only be inserted with $-\delta = w_i - w_{i-1}$ and $\delta = w_{j+1} - w_j$, so $w_{i-1} - w_i = w_{j+1} - w_j$, in which case it is easy to see that $F_{\boldsymbol{w}}$ is reduced by 2. Furthermore, if $\delta < 0$ and we had some $k \in [i, j]$ with $-u_k > \delta$, the k-th iteration would have deleted δ from E. This means that (i, j, δ) is indeed an event that reduces $|F_{\boldsymbol{w}}|$ and does not make any u_k drop to 0.

Reciprocally, if there is an event (i, j, δ) to be found we want to prove that the algorithm returns something (not necessarily the same event). If the algorithm exits before iteration j, it returns some event that we have already proven must be correct. Let us assume that we do not exit the loop before iteration j : we have added $-\delta$ at rank i, and it is still in R because for every $k \in [i, j]$ we did not have $-\delta > u_k$ by hypothesis. Since $-\delta$ is in E and $w_{j+1} - w_j = x$, the algorithm returns (i, j, δ).

Complexity. The complexity of *findGoodEvent* depends on the following operations: we need to be able to test the existence of a value in a dictionary, to add a key/value pair and, a bit less usual, to filter all values lower than a certain amount (the last line of *findGoodEvent*). We can use a *treap* structure (see [1]), which is a form of binary search tree that allows to split the values higher and lower to a certain number in $\log n$ time. This gives us a total complexity of $\mathcal{O}(n \log(n))$.

References

[1] Raimund Seidel and Cecilia R Aragon. Randomized search trees. *Algorithmica*, 16(4-5):464–497, 1996.
