# Supplementary material for: Comparing copy-number profiles under multi-copy amplifications and deletions
Source: BMC Genomics. 2020 Apr 16;21(Suppl 2):198. doi: 10.1186/s12864-020-6611-3 (PMC7160889; doi:10.1186/s12864-020-6611-3)
Supplement: Supplementary file 2 — Additional file 2 Supplementary file S33-S2.pdf contains all the additional experimental results. [file 12864_2020_6611_MOESM2_ESM.pdf]

Comparing Copy-Number Profiles Under Multi-Copy Amplifications and Deletions - Supplementary Material II Additional experimental results

For each combination of values of $r \in \{0.01, 0.05, 0.1\}$ and $q \in \{0.25, 0.5, 0.75, 1\}$, we ran four sets of experiments, each one isolating one of the l, n, Δ and (e_{min}, e_{max}) parameters (as in the main text). We present the result obtained for each possible r and q on error-free data.

We then show the obtained results on noisy data, again for each possible r and q. For each error rate $\alpha \in \{0, 0.1, 0.25, 0.5, 1\}$, we repeated the same scheme of isolating every parameter. The number of combinations of the parameters r, q, α is 60, and for each combination we again isolated one of l, n, Δ and (e_{min}, e_{max}). We do not list every plot here: we show the results of error rates in $\{0.1, 0.25, 0.5\}$ for default r and q (we omit error rates higher than 1, as they always result in bad trees). All the missing plots can be accessed through the public data directories (download instructions are provided on the git readme file of the cnp2cnp project).

We note that all the experiments can be reproduced by downloading the git project and executing the command

```
> python3 autosimulation.py
```

This python script generates trees, infers reconstructions and evaluates them for all possible parameter values. Expect 1-3 weeks of running time on a regular PC.

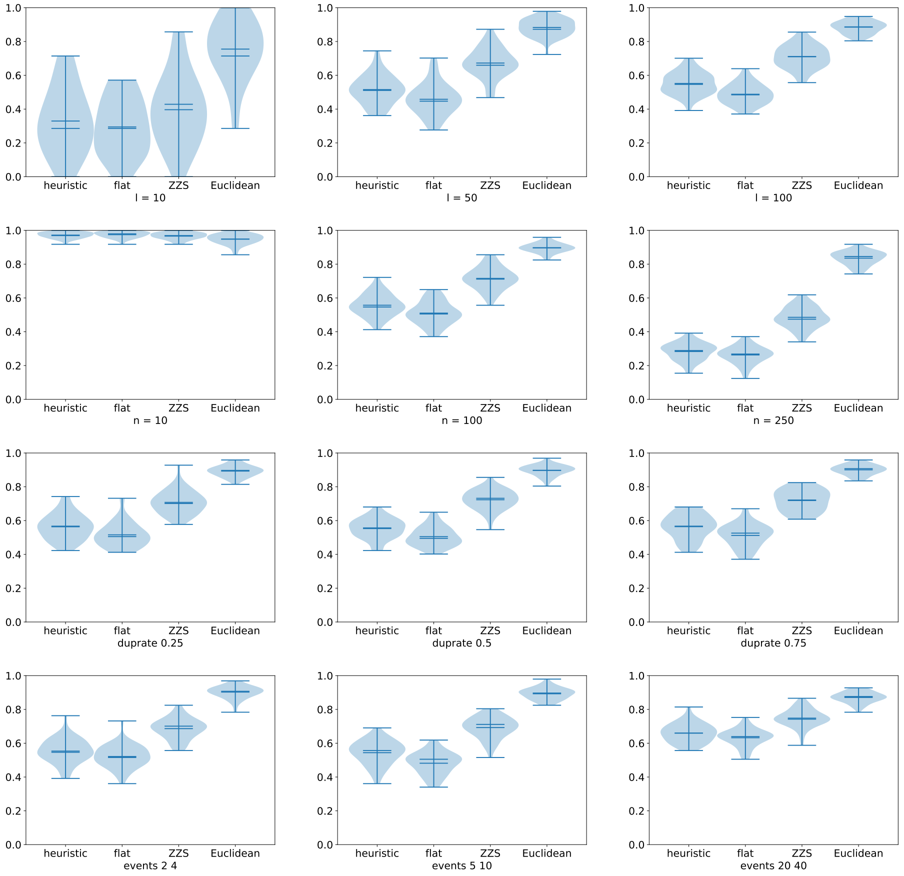

Figure 1: Violin plots for $r = 0.01, q = 0.25$ (error-free data).

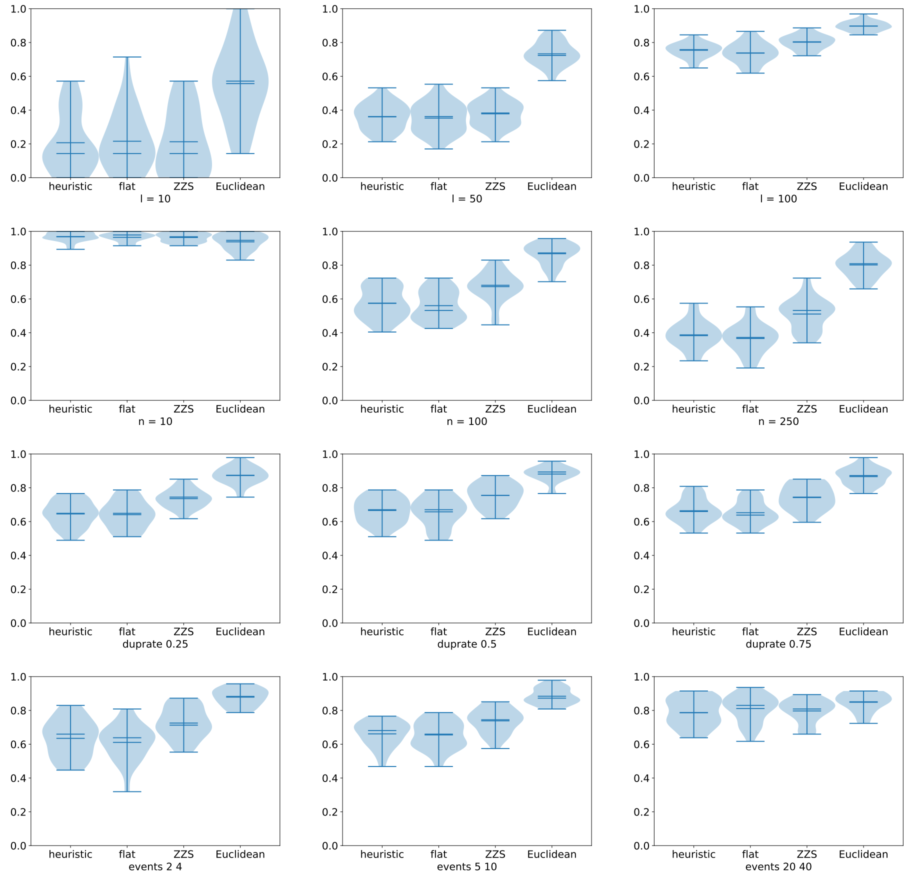

Figure 2: Violin plots for $r = 0.01, q = 0.5$ (error-free data).

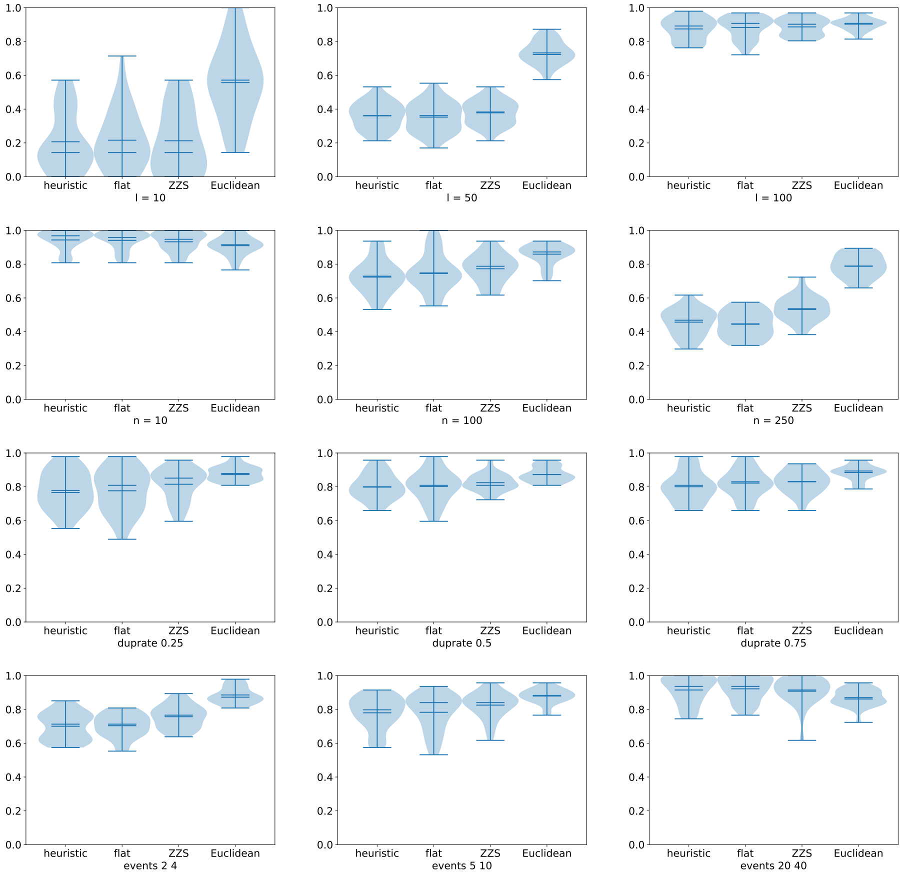

Figure 3: Violin plots for $r = 0.01, q = 0.75$ (error-free data).

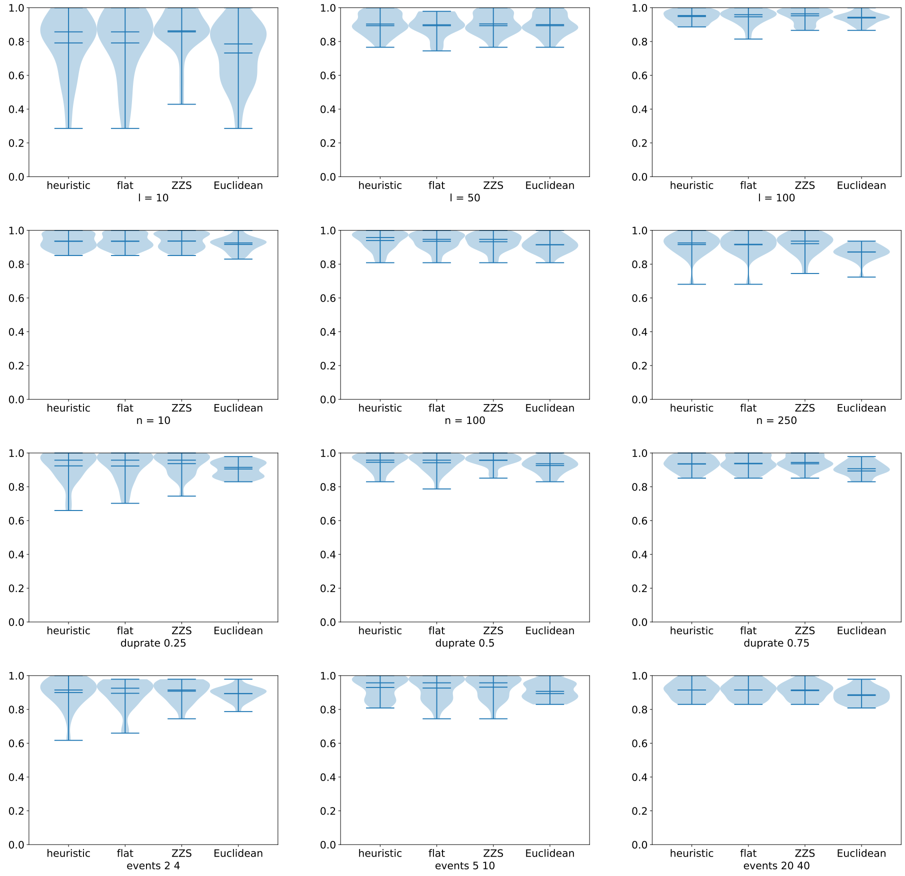

Figure 4: Violin plots for $r = 0.01, q = 1$ (error-free data).

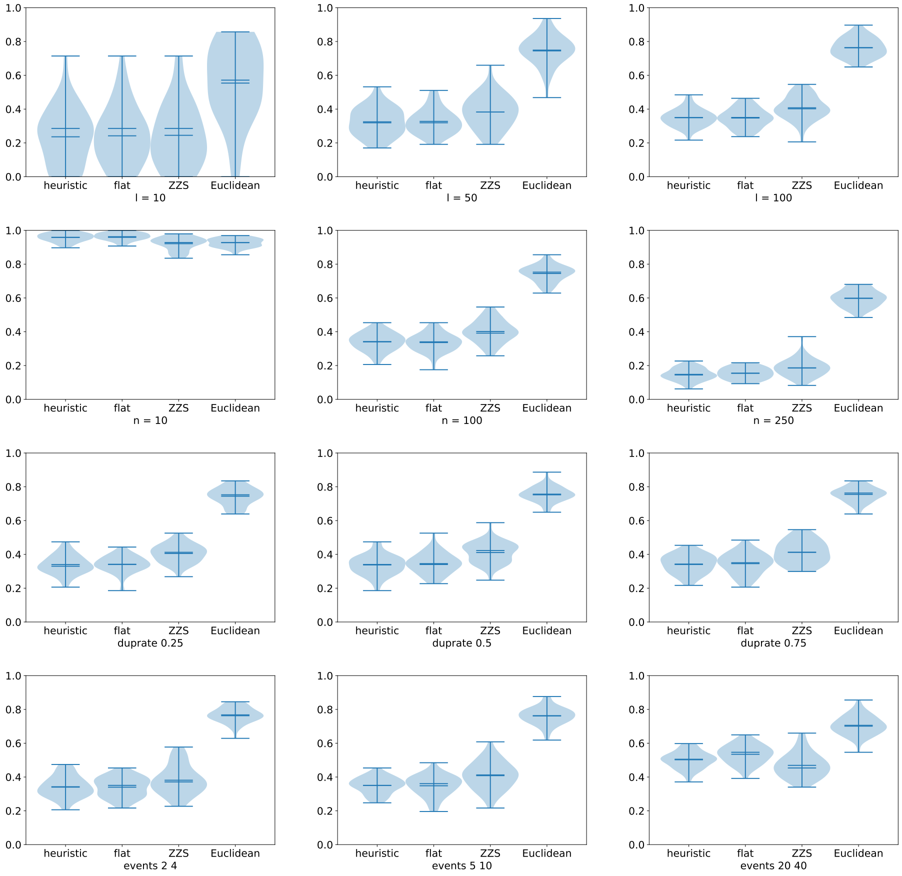

Figure 5: Violin plots for $r = 0.05, q = 0.25$ (error-free data).

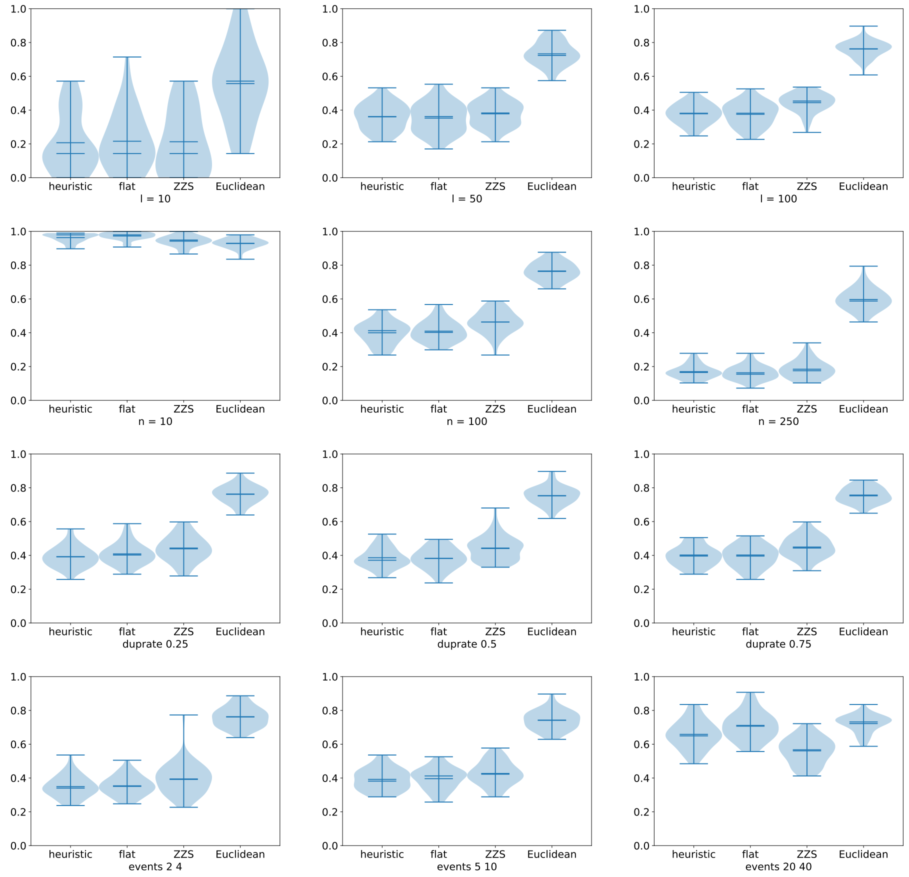

Figure 6: Violin plots for $r = 0.05, q = 0.5$ (error-free data).

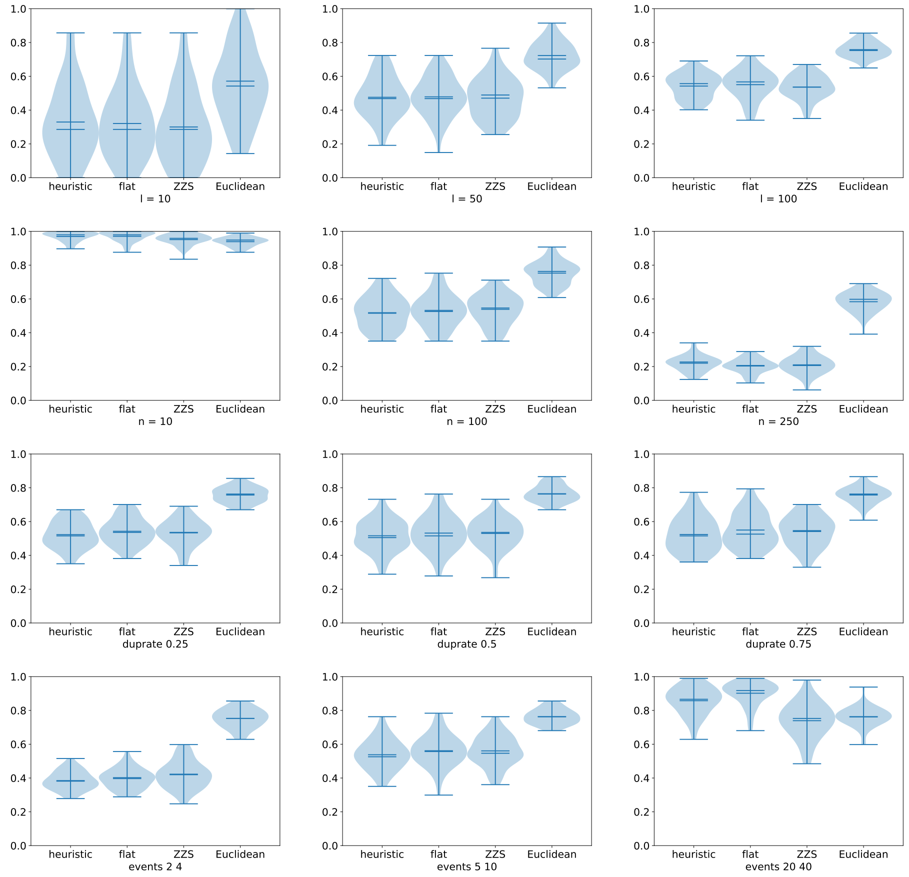

Figure 7: Violin plots for $r = 0.05, q = 0.75$ (error-free data).

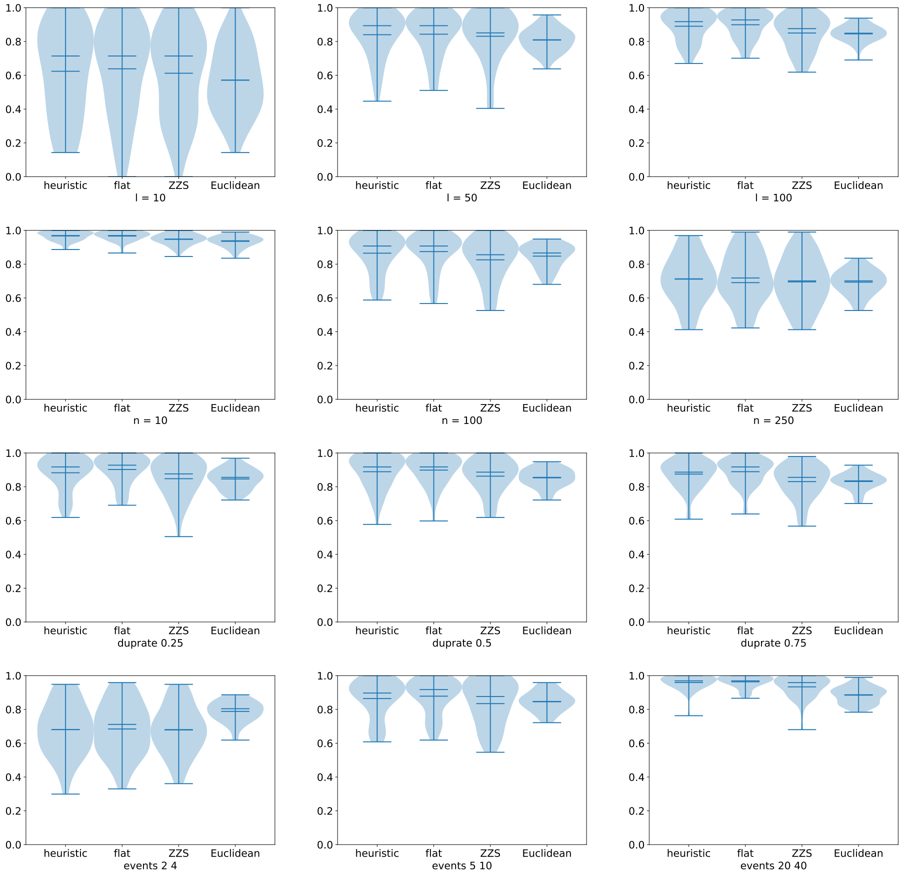

Figure 8: Violin plots for $r = 0.05, q = 1$ (error-free data).

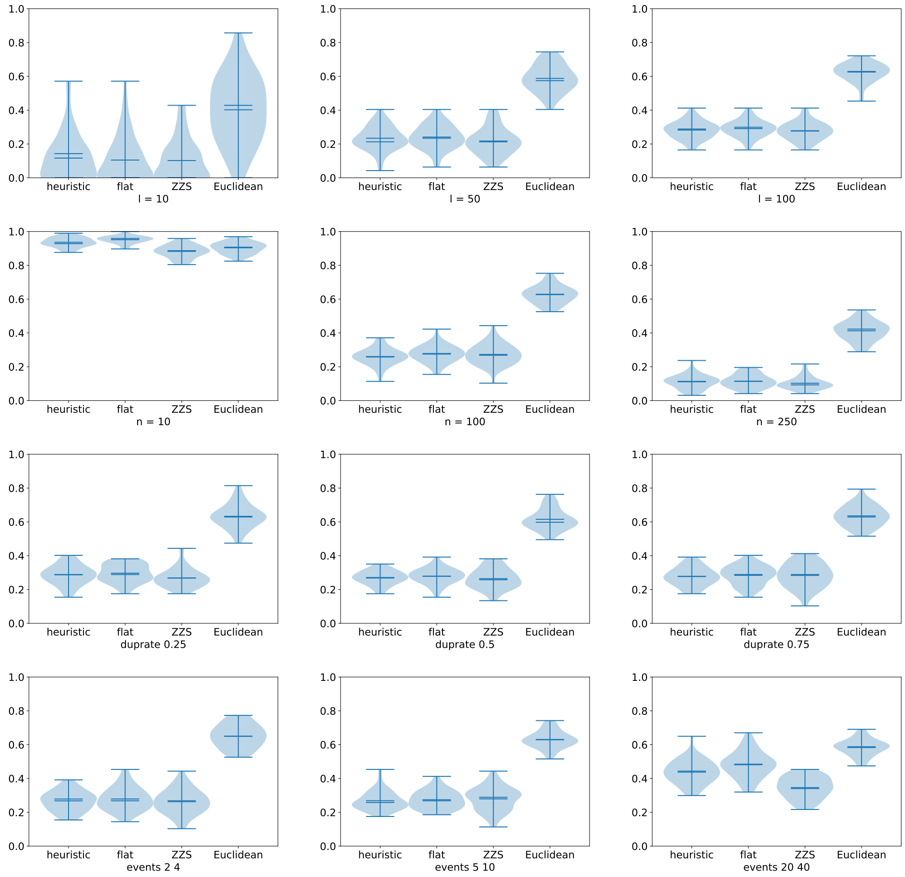

Figure 9: Violin plots for $r = 0.1, q = 0.25$ (error-free data).

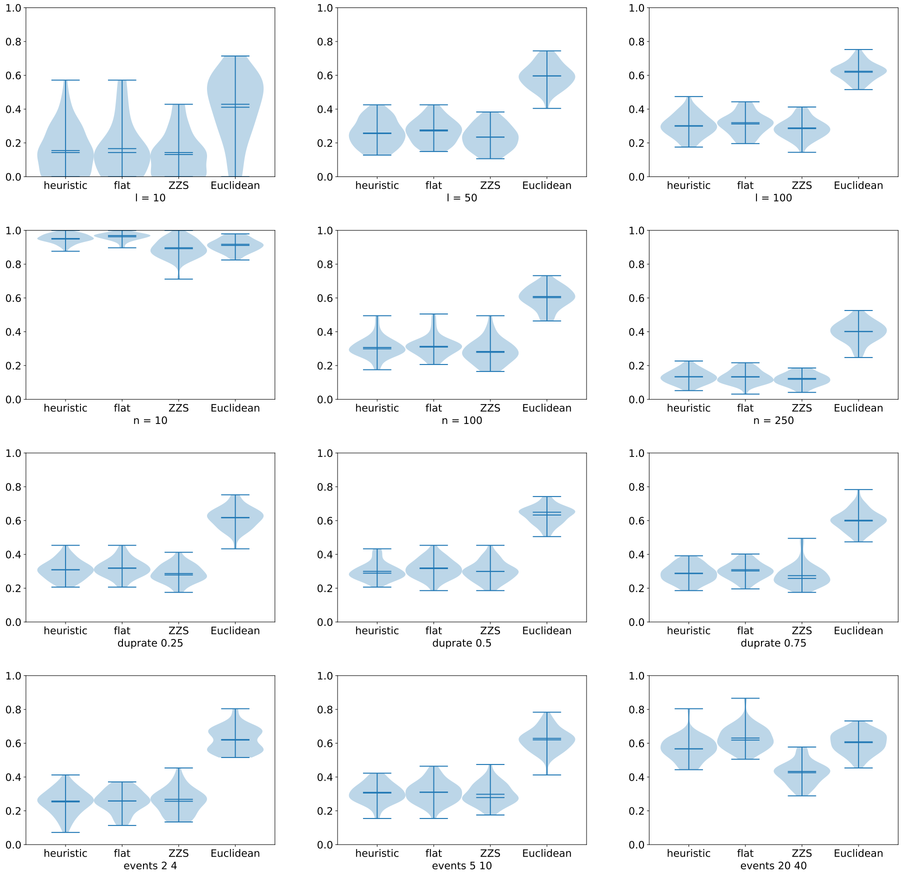

Figure 10: Violin plots for $r = 0.1, q = 0.5$ (error-free data).

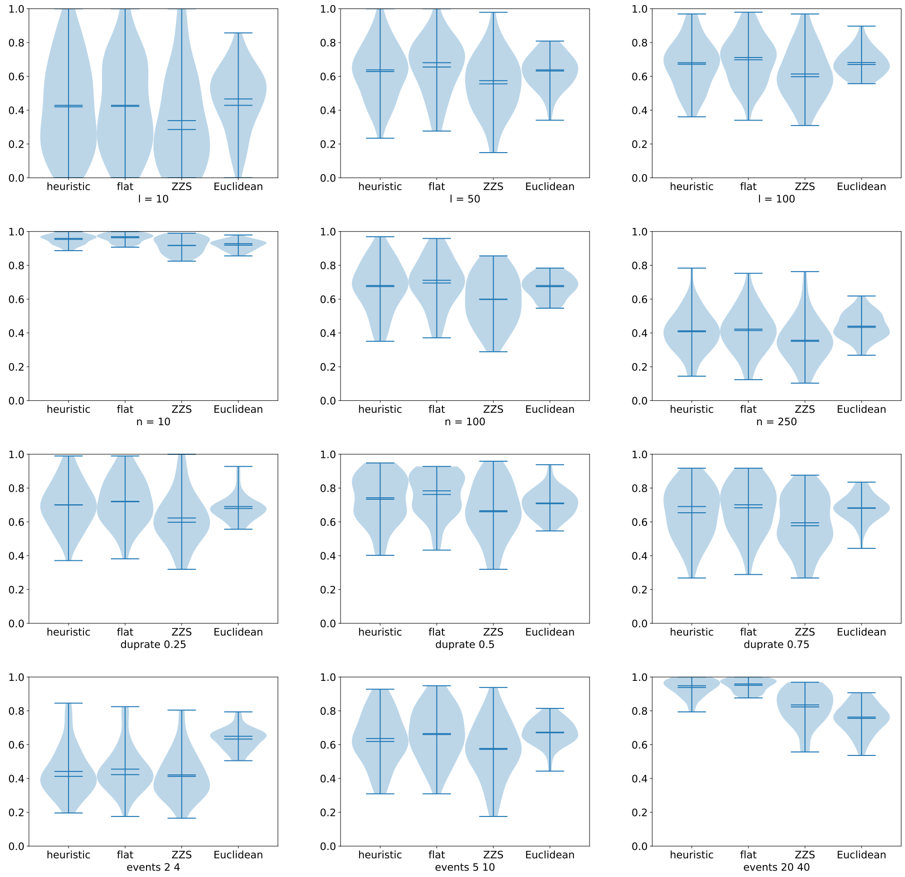

Figure 11: Violin plots for $r = 0.1, q = 0.75$ (error-free data).

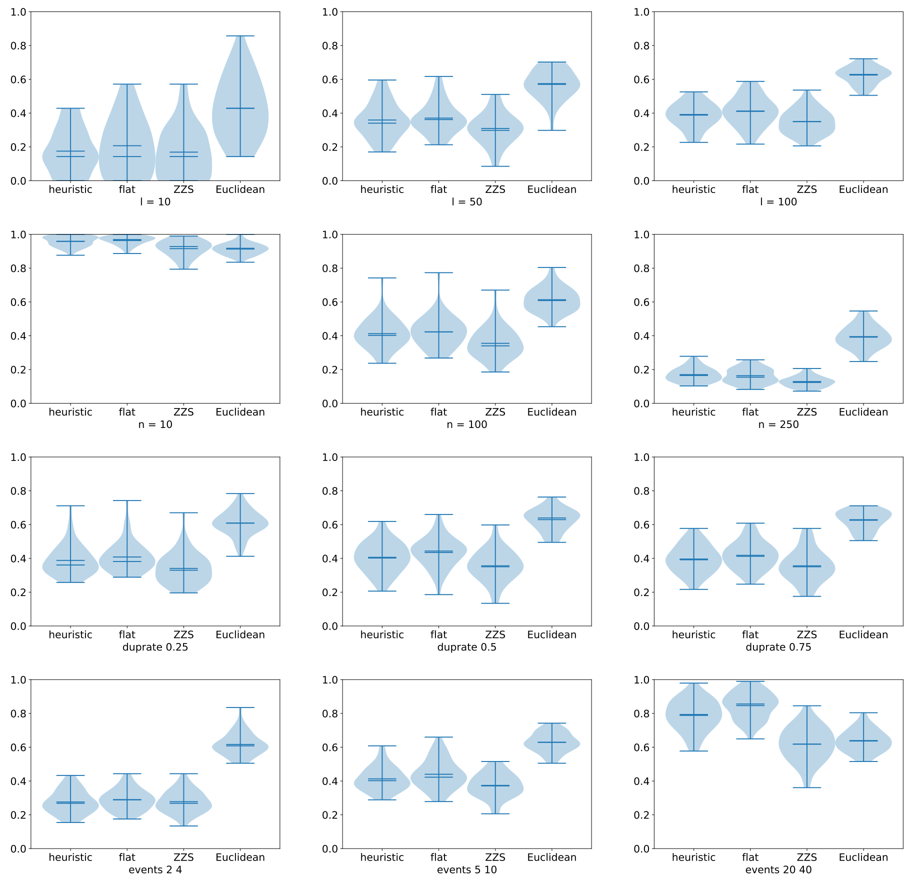

Figure 12: Violin plots for $r = 0.1, q = 1$ (error-free data).

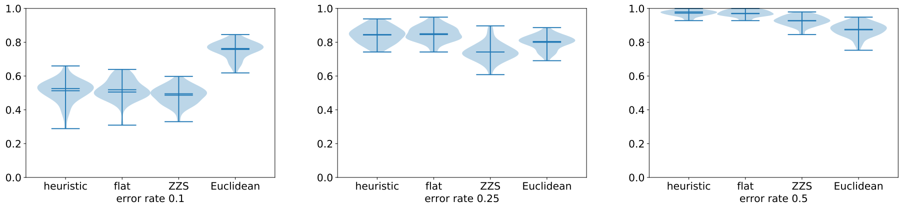

Figure 13: Noisy data with $r = 0.05$ and $q = 0.25$.

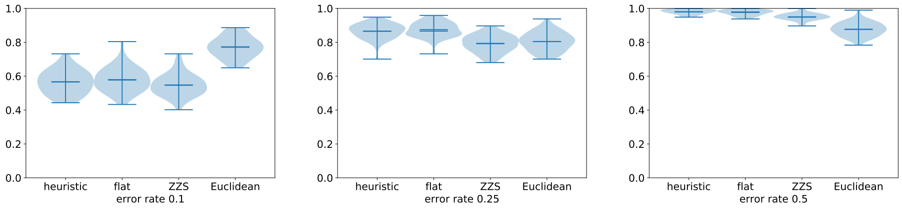

Figure 14: Noisy data with $r = 0.05$ and $q = 0.5$.

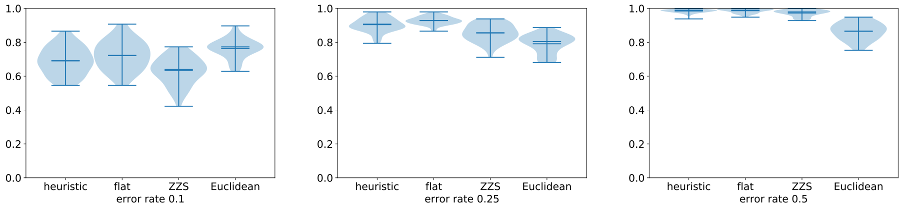

Figure 15: Noisy data with $r = 0.05$ and $q = 0.75$.

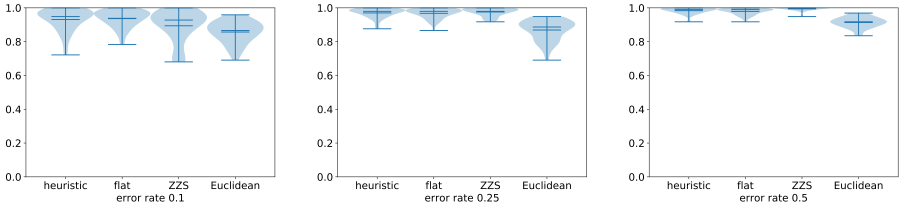

Figure 16: Noisy data with $r = 0.05$ and $q = 1$.

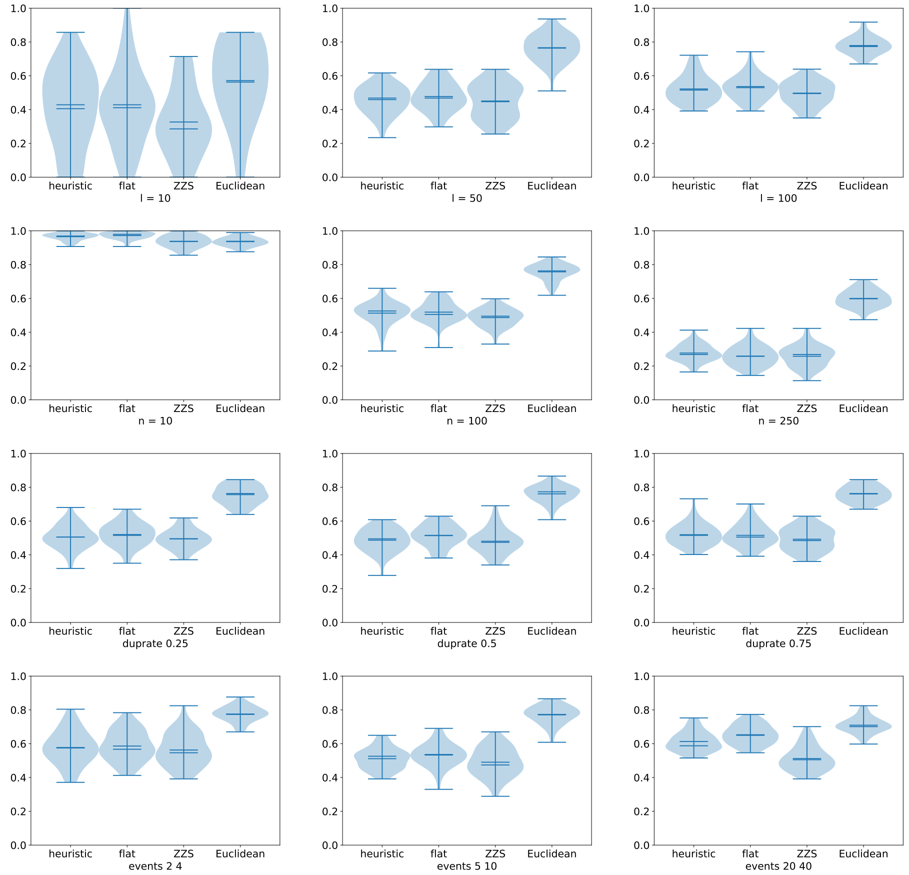

Figure 17: Violin plots for $r = 0.05, q = 0.25$ and error rate $\alpha = 0.1$.

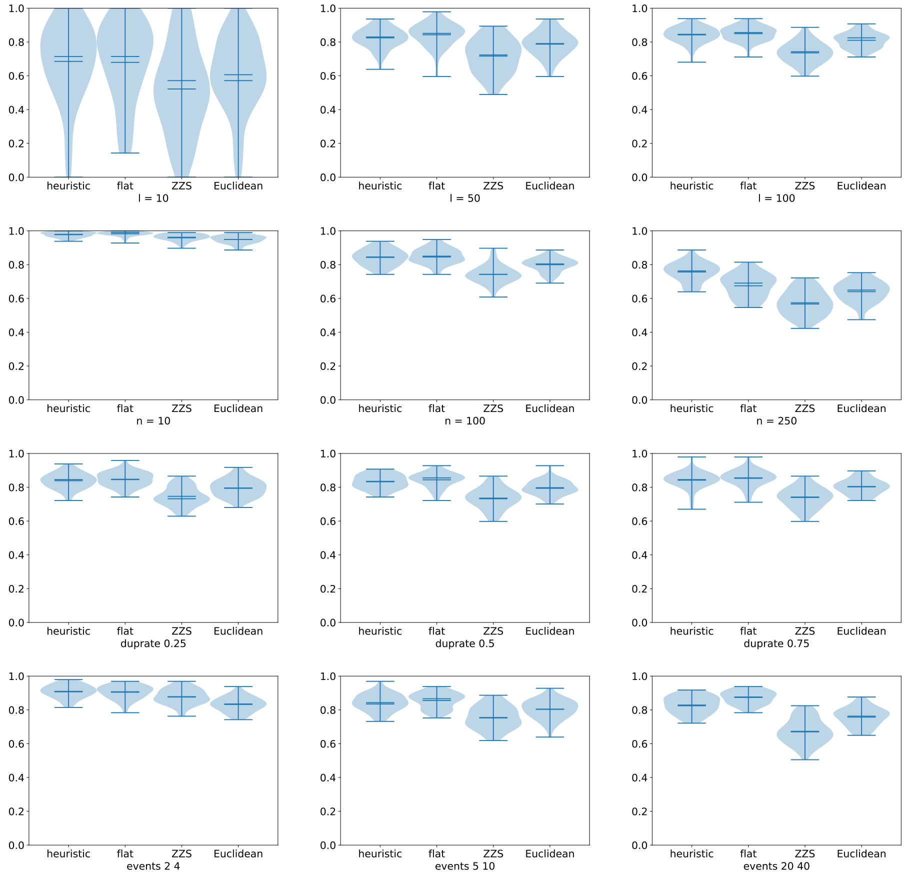

Figure 18: Violin plots for $r = 0.05, q = 0.25$ and error rate $\alpha = 0.25$.

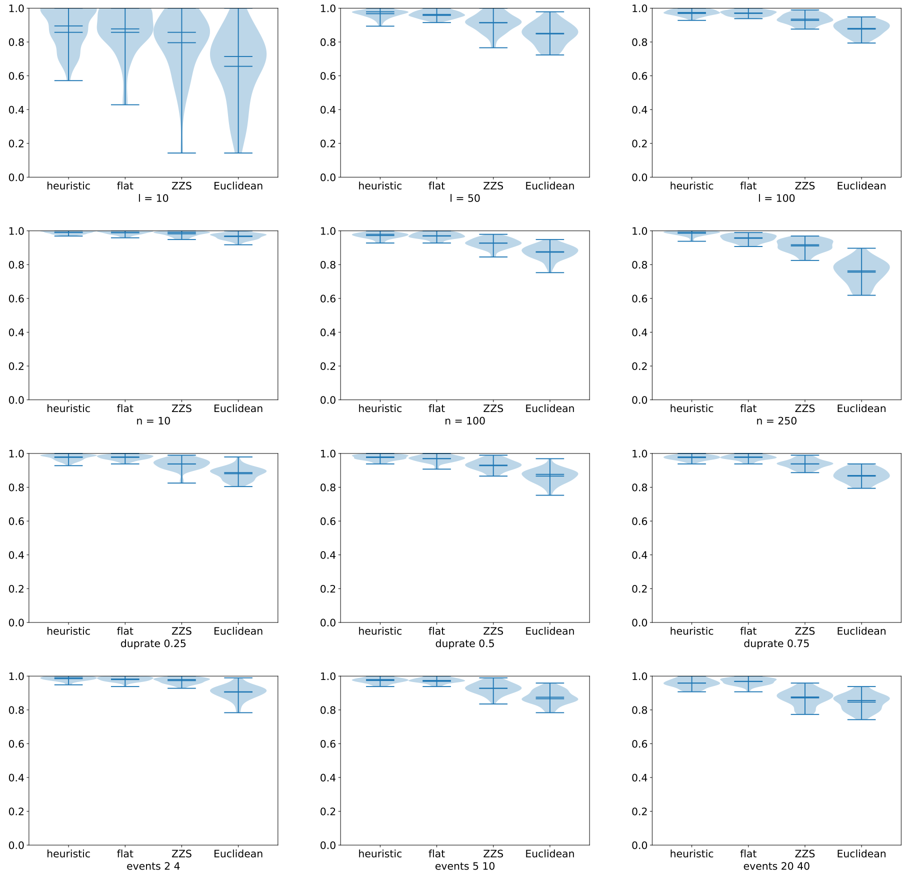

Figure 19: Violin plots for $r = 0.05, q = 0.25$ and error rate $\alpha = 0.5$.
